# Suppression of CD13 Enhances the Cytotoxic Effect of Chemotherapeutic Drugs in Hepatocellular Carcinoma Cells

**DOI:** 10.3389/fphar.2021.660377

**Published:** 2021-05-11

**Authors:** Shengping Ji, Yuqian Ma, Xiaoyan Xing, Binbin Ge, Yutian Li, Xinyue Xu, Jiliang Song, Mei Xiao, Feng Gao, Wenyan Jiang, Chunyan Fang, Xuejian Wang

**Affiliations:** ^1^ School of Pharmacy, Weifang Medical University, Weifang, China; ^2^ Health Management Center, Weifang People’s Hospital, Weifang, China

**Keywords:** heptaocellular carcinoma, CD13, ROS, ubenimex, multidrug resistance

## Abstract

Multidrug resistance (MDR) of hepatocellular carcinoma (HCC) is a serious problem that directly hinders the effect of chemotherapeutics. In this study, we mainly explore the molecular mechanism of ROS-induced CD13 expression using hepatocarcinoma cells as the research object. We show that the drug of fluorouracil (5FU), epirubicin (EPI) and gemcitabine (GEM) can induce ROS generation, activate Ets2 and promote CD13 expression. Meanwhile, CD13 can activate NRF1 and up-regulate ROS scavenging genes transcription, such as SOD1, GPX1, GPX2 and GPX3, leading to down-regulation of intracellular ROS level and reducing the sensitivity of cells to chemotherapy agent. We also detected the anti-tumor effect of the combination therapy, CD13 inhibitor ubenimex and a variety of conventional anti-cancer drugs, such as 5FU, EPI, GEM, pemetrexed (Pem) and paclitaxel (PTX) were employed in combination. Ubenimex enhances the sensitivity of different chemotherapeutic agents and cooperates with chemotherapeutic agents to suppress tumor growth *in vitro* and *in vivo*. In general, overexpression of CD13 can lead to chemotherapy resistance, and CD13 inhibitor can reverse this effect. Combination of chemotherapy agent and ubenimex will become a potential treatment strategy for liver cancer resistance.

## Introduction

Hepatocellular carcinoma (HCC) is one of the most prevalent malignancies in worldwide, and its morbidity and mortality rates have been increasing in recent years (Mlynarsky et al., 2015). Due to inoperable or postoperative recurrence and other reasons, chemotherapy is still an important treatment for patients with HCC. However, multidrug resistance is an important reason for treatment failure.

Aminopeptidase N (APN/CD13) is a 150 kD zinc-bound type 2 transmembrane peptidase, which forms a non-covalently bound homodimer on the cell membrane (Mina-Osorio, 2008). It is widely expressed in mammalian cells, such as renal proximal tubular epithelial cells and myeloid progenitor cells, as well as intestinal epithelial and central nervous system synaptic membranes. As a multifunctional protein, APN/CD13 plays an important role in viral receptor function, cell differentiation and antigen presentation, as well as tumor invasion, metastasis, angiogenesis and anti-apoptosis (Dixon et al., 1994; Fukasawa et al., 2006). The expression of APN/CD13 in lung cancer, pancreatic cancer, colon cancer and acute lymphoblastic leukemia has been reported (Dalal et al., 2014). Reactive oxygen species (ROS) are generally considered by-products of oxygen consumption and cellular metabolism, formed by the partial reduction of molecular oxygen (Zorov et al., 2014). ROS have long been associated with cancer where different types of tumor cells have been shown to produce elevated level of ROS compared to their normal counterparts(Panieri and Santoro, 2016). Elevated level of ROS are thought to be oncogenic, causing damage to DNA, proteins and lipids, promoting genetic instability and tumourigenesis (Liou and Storz, 2010). ROS also act as signalling molecules in cancer, contributing to abnormal cell growth, metastasis, resistance to apoptosis, angiogenesis and differentiation block in some types of cancer (Sabharwal and Schumacker, 2014). Increased ROS level are pro-tumourigenic, resulting in the activation of pro-survival signalling pathway, loss of tumour suppressor gene-function, increased glucose metabolism, adaptation to hypoxia and the generation of oncogenic mutation (Levine and Puzio-Kuter, 2010). However, toxic level of ROS production in cancer are anti-tumourigenic resulting in an increase of oxidative stress and induction of tumour cell death (Nogueira et al., 2008). For this reason, therapy used to eliminate or elevate ROS production may be potentially effective, although it is a rather challenging concept. We have previously reported that CD13 inhibitor, neutralizing antibody, and CD13-targeted siRNA could up-regulate intracellular ROS level and enhance the anti-tumor effect of chemotherapy agents (Zhang et al., 2018).

Therefore, in this study we explored the relationship between ROS generation and CD13 expression. we confirmed that chemotherapeutics up-regulated ROS generation and activated Ets2 expression, a transcription factor in upstream of CD13 and promote CD13 expression. After CD13 overexpression, reactive oxygen species scavenging genes were up-regulated. After intracellular ROS generation, it would feedback up-regulation of CD13 and promote the expression of genes that eliminate ROS, thereby down-regulating ROS level to reduce the sensitivity of cells to chemotherapeutics. Ubenimex, a CD13 inhibitor, could inhibit this effect and partially revert chemotherapy resistance of HCC.

## Meterials and Methods

### Cell Culture and Reagents

Human liver cancer cells PLC/PRF/5 and Huh7, human lung adenocarcinoma cells A549 and human breast cancer cells MDA-MB-231, were obtained from the Cell Bank of Shanghai (Shanghai, China). PLC/PRF/5 and Huh7 were cultured in modified Eagle’s medium (MEM) and Dulbecco’s modified Eagle's medium (DMEM) respectively with 10% fetal bovine serum (FBS). A549 and MDA-MB-231 were cultured in Roswell Park Memorial Institute (RPMI)-1640 supplemented with 10% fetal bovine serum (FBS). All cell lines were cultured at 37°C in a humidified atmosphere of 5% CO_2_. We constructed stable CD13 overexpression (PLC/PRF/5/CD13) and knockdown (PLC/PRF/5/CD13 short hairpin RNA) PLC/PRF/5 cells by using the lentiviral vector (Genechem, China) and puromycin screening (Zhang et al., 2018).

Lipofection 2000 was purchased from Invitrogen (Cat. 11668–019). siRNA was synthesized by Shanghai GenePharma. Ubenimex (Cat. B8385), 5-Fluorouracil (5FU, Cat. F6627) and 2′, 7′-Dichlorofluorescin diaceta (DCFH-DA) were purchased from Sigma. Gemcitabine (GEM, Cat. G8970), Paclitaxel (PTX, Cat. SP8020), Pemetrexed (Pem, Cat. SP9860) and doxorubicin (Epirubicin Hydrochloride, Cat. IE0640) were purchased from Solarbio. N-acetyl-l-cysteine (NAC, Cat. S0077) was purchased from Beyotime.

### Cellular Reactive Oxygen Species Detection

The level of intracellular ROS was quantified using a fluorescent probe 2′, 7′-dichloro-cluorescein diacetate (DCFH-DA). Cells were seeded in 6-well plates (5 × 10^^5^ cells/well) and treated with drugs for 48 h. Then, cells were harvested and incubated with 10 μM DCFH-DA for 20 min at 37°C in the dark. After incubation, the cells were washed three times with PBS and analyzed by flow cytometry.

### Determination of CD13 Expression by Flow Cytometry

The cells were seeded in 6-well plates (5 × 10^^5^ cells/well) and treated with drugs for 48 h, cells were harvested and incubated with PE-conjugated monoclonal antibody targeting CD13 (BD Pharmingen, CD13mAb clone:WM15) for 60 min on ice. Then, the cells were analyzed by flow cytometry.

### Mitochondrial Membrane Potentials (ΔΨm)

The ΔΨm changes of treated spermatozoa were evaluated using JC-1 (lipophilic cation 5, 5, 6, 6′-tetrachloro-1, 1′, 3, 3′-tetraethylbenzimidazolcarbocyanine iodide) Mitochondrial Membrane Potential Detection Kit (Beyotime Institute of Biotechnology, Haimen, China). JC-1 is capable of selectively entering mitochondria where it forms monomers and emits green fluorescence when ΔΨm is relatively low. At high ΔΨm, JC-1 aggregates and gives a red fluorescence. Assays was initiated by incubating PLC/PRF/5 cells with JC-1 for 20 min at 37°C in the dark and the fluorescence of separated cells was detected by flow cytometer.

### Cell Proliferation Assays

The PLC/PRF/5, Huh7, A549 and MDA-MB-231 cells were seeded at a density of 5 × 10^^3^ cells/well in 96-well plates. The EdU kit (cat. C0088L; Beyotime Institute of Biotechnology, Shanghai, China) was used to detect cell proliferation according to the manufacturer's instruction. The cells were treated with drugs for 48 h, washed with PBS, and incubated with 10 μM EdU in culture medium at room temperature for 2 h. The resulting absorbance was measured at 450 nm on a spectrophotometer. Each treatment was performed in triplicate wells per experiment.

### Bioinformatic Data Mining

We selected the liver cancer samples after chemotherapy from the TCGA data (https://www.home-for-researchers.com/static/index.html#/) and analyzed the correlation between the expression of CD13 and Ets2. ANPEP indicates CD13.

### Apoptosis Analysis

PLC/PRF/5 cells were seeded in 6-well plate at the concentration of 5 × 10^^5^ cells/well and treated with drugs for 24 h. Next, cells were collected and stained using Annexin V-FITC/PI kit (Best Biotech, China) according to the manufacturer’s instructions and analyzed using a flow cytometer with Cell Quest Software (FACSCalibur, BectonDickinson, United States).

### Immunoblotting Analysis

The cells were lyzed in RIPA lysis buffer containing protease inhibitor cocktail (Sigma–Aldrich) for 30 min on ice. Protein concentration was measured using a BCA protein assay kit (Beijing Solarbio Science and Technology). Then 30 μg proteins were electrophoresed, separated in SDS-PAGE and transferred onto polyvinylidene difluoride (PVDF) membrane (Millipore). The target proteins were determined Immunoblotting with their respective specific antibodies. *β*-actin or total protein was used as an internal control. Blots were visualized by an enhanced chemiluminescence kit (Millipore). Densitometry for Immunoblotting was performed by using AlphaEaseFC-v4.0.0 software. The following antibodies were used in this study: CD13 (dilution of 1:1000, catalog number sc-51522; Santa Cruz Biotechnology, China), Ets2 (dilution of 1:1000, catalog number sc-365666; Santa Cruz Biotechnology, China), NRF1 ((dilution of 1:1000, catalog number 46743; Cell Signaling Technology), and *β*-actin (dilution of 1:1000, catalog number sc-1616; Santa Cruz Biotechnology).

### Ribonucleic Acid Sequencing

Total RNA was extracted with Trizol reagent (Ambion, United States), then the library construction and RNA-sequencing (RNA-seq) were performed at Shanghai Genechem (Shanghai, China) with Illumina NovaSeq 6000 (Illumina, United States), followed by the computational analysis.

### siRNA Transfection

The RNA interference technique was used to downregulate Ets2 and NRF1 in PLC/PRF/5 and Huh7 cells. The sequences were as follows: siEts2#1, 5′-GAG​ACG​GAU​GGG​AGU​UUA-3′; siEts2#2, 5′-AAC​AGU​UAC​AGA​GGG​ACA​CUC​CCU​GUC​UC-3′; and siNRF1#1, 5′-TTA​GGG​TTT​GAA​GCT​CGA​TAT-3′; siNRF1#2, 5′-CACAUUGGCUGAUGCUUCAdTdT-3; negative control (NC) siRNA, 5′-UUC​UCC​GAA​CGU​GUC​ACG​UTT-3′.

siRNA (100 pmol) was transfected using Lipofectamine®2000 (5 µL) transfection reagent (Life Technologies, United States) and serum-free MEM and DMEM in a six-well plate. 6 h after transfection, siRNA was removed by replacing the medium. After 48 h of transfection, we performed qRT-PCR or immunoblotting to determine the knockdown efficiency.

### Quantitative Real-Time Polymerase Chain Reaction

Total RNA was extracted with Trizol reagent (Ambion, United States), and the RNA concentration was detected by a NanoDrop instrument. Single-strand cDNA was reverse-transcribed using a HiFiscript cDNA Synthesis Kit (CWBIO, China) according to the manufacturer’s instruction. The cDNA was then amplified with a SYBR Green mixture (CWBIO, China) and gene-specific primers. The primers used for qRT-PCR were Table 1. Finally, qRT-PCR was performed on an Authorized Thermal Cycler (LighterCycler480 Systems, United States) and the relative mRNA level of the target gene was measured via the 2^−ΔΔCT^ method. GAPDH served as an internal control, and each reaction was performed in triplicate.

**TABLE 1:** Sequences of primers used for mRNA detection by qRT-PCR.

Gene		Primer sequence (5'-3')
CD13	Forward	5′-GCC​CAC​CTG​GAA​CTT​GAA​AG
	Reverse	5′-AGA​TGG​CGT​CAA​ACA​GCT​CA-3′
Ets2	Forward	5′-CTG​ACT​TTG​TGG​GTG​TTC​TCT​GG-3′
	Reverse	5′-GGA​ACG​GAG​GTG​AGG​TGT​GAA​TTT​TC-3′
NRF1	Forward	5'-GAC​TCG​CCT​TCT​TCT​CCC​G-3'
	Reverse	5'-GGG​TTA​GGT​TTG​GAG​GGT​GA-3′
SOD1	Forward	5′-AAG​GCC​GTG​TGC​GTG​CTG​AA-3′
	Reverse	5′-GGC​CCA​CCG​TGT​TTT​CTG​GA-3′
GPX1	Forward	5′-CCA​AGC​TCA​TCA​CCT​GGT​CT-3′
	Reverse	5′-TCG​ATG​TCA​ATG​GTC​TGG​AA-3′
GPX2	Forward	5′-GGT​AGA​TTT​CAA​TAC​GTT​CCG​GG-3′
	Reverse	5′-TGA​CAG​TTC​TCC​TGA​TGT​CCA​AA-3′
GPX3	Forward	5′-GGG​GAT​GTC​AAT​GGA​GAG​AA-3′
	Reverse	5′-TTC​ATG​GGT​TCC​CAG​AAG​AG-3′

### stubRFP- sensGFP-LC3B System

The PLC/PRF/5 cells were infected with the stubRFP-sensGFP-LC3B lentivirus (Genechem, Cat # GRL 2001, China) and screened by puromycin (8 μg/ml). The fluorescence of the construct depended on the difference in pH between the acidic autolysosome and the neutral autophagosome. The progression of autophagic flux was assessed by monitoring the red/green (yellow) or red fluorescence of the cells. The infected cells were treated with 5FU/Ubenimex, GEM/Ubenimex and EPI/Ubenimex for 24 h and their fluorescence was detected by laser scanning confocal microscopy.

### Xenograft Model Assay

Four week-old female Kunming mice were purchased from Hunan SJA Laboratory Animal Company (Hunan, China) and fed under specific pathogen-free conditions. H22 cells were collected and resuspended at a density of 5 × 10^^7^/ml with PBS. Then, 200 μl cell suspension was subcutaneously injected into the mice. After tumor implantation, the mice were randomly divided into five groups (*n* = 8) and administered PBS or Pem (150 mg/kg/week), GEM (50 mg/kg/3 days) and ubenimex (74 mg/kg/d).Body weights and tumor volumes were monitored every 3 days. After 3 weeks, all mice were sacrificed and dissected, and tumor tissue, liver and spleen tissue were weighed. All animal experiments were approved by the Guidelines of the Animal Care and Use Committee of Weifang Medical University.

### Statistical Analysis

Data was presented as the mean ± SEM, and analyzed by Student’s t-test or one-way ANOVA. Multiple comparison between the groups was performed using S-N-K method. Statistical significance was set at a level of *p* < 0.05. Statistical analysis was done with SPSS/Win11.0 software (SPSS, Inc., Chicago, Illinois).

## Results

### Chemotherapy Drug Damages Mitochondria and Upregulates Reactive Oxygen Species Level to Induce CD13 Expression

**FIGURE 1:**
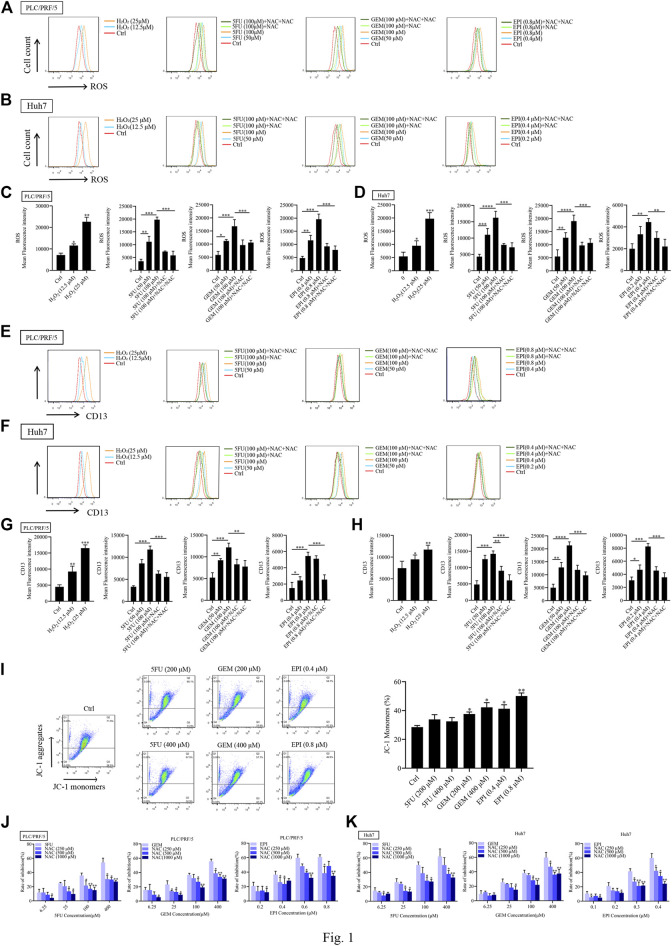
Chemotherapeutic drugs upregulates ROS and induce CD13 expression. Effect of chemotherapeutic drugs on ROS levels. PLC/PRF/5 and Huh7 cells were treated with H_2_O_2_ or 5FU, GEM and EPI combined with NAC (500 μM) for 48 h. **(A–D)** The intracellular ROS levels **(E–H)** the expression of CD13 detected by flow cytometry. Data are presented as mean ± SEM (*n* = 3). *p ≤ 0.05, ***p ≤ 0.001. **(I)** The mitochondrial membrane potential measured by flow cytometry in PLC/PRF/5 cells treated with 5FU, GEM, and EPI. Data are presented as mean ± SEM (*n* = 3). *p ≤ 0.05, **p ≤ 0.01. **(J,K)** Cell proliferation was measured by EdU assay in PLC/PRF/5 and Huh7 cells treated with 5FU, GEM and EPI, or combined with NAC for 48 h. Data are presented as mean ± SEM (*n* = 3). *p ≤ 0.05; **p ≤ 0.01.

To determine whether ROS induces CD13 expression, PLC/PRF/5 and Huh7 cells were treated with different concentrations of hydrogen peroxide (H_2_O_2_), then the intracellular ROS level and CD13 expression were detected by flow cytometry. It was found that H_2_O_2_ treatment raised ROS level and CD13 expression with significant difference, indicating that ROS is involved in the expression of CD13. To further verify the above conjecture, chemotherapy drugs 5FU, GEM and EPI were employed to treat PLC/PRF/5 and Huh7 cells in combination with the reactive oxygen scavenger NAC. We found that the intracellular ROS level and CD13 expression were significantly upregulated in single chemotherapeutic drugs group. However, after 1h of pretreatment with NAC and 48h of treatment with chemotherapy drugs, the intracellular ROS and CD13 levels were significantly lower than those in chemotherapy drugs alone (Figures 1A–H).

We also found that the mitochondrial membrane potential of cells treated with chemotherapeutic drug decreased significantly (Figure 1I), indicating that chemotherapeutic drug damaged mitochondria and released ROS. In addition, EdU assay was used to detect the cytotoxicity of three chemotherapeutic drugs combined with NAC, the data showed that the cytotoxicity was enhanced with the increasement of chemotherapeutic drug concentration. Then the cells were treated with chemotherapeutic drugs in combination with different concentrations of NAC. The addition of NAC could significantly reduce the cytotoxicity of chemotherapeutic drug (Figures 1J,K). These results indicated that chemotherapeutic drug induces CD13 expression by up-regulation of intracellular ROS level.

### Reactive Oxygen Species Promotes CD13 Expression by Activating Transcriptional Factor Ets2

**FIGURE 2:**
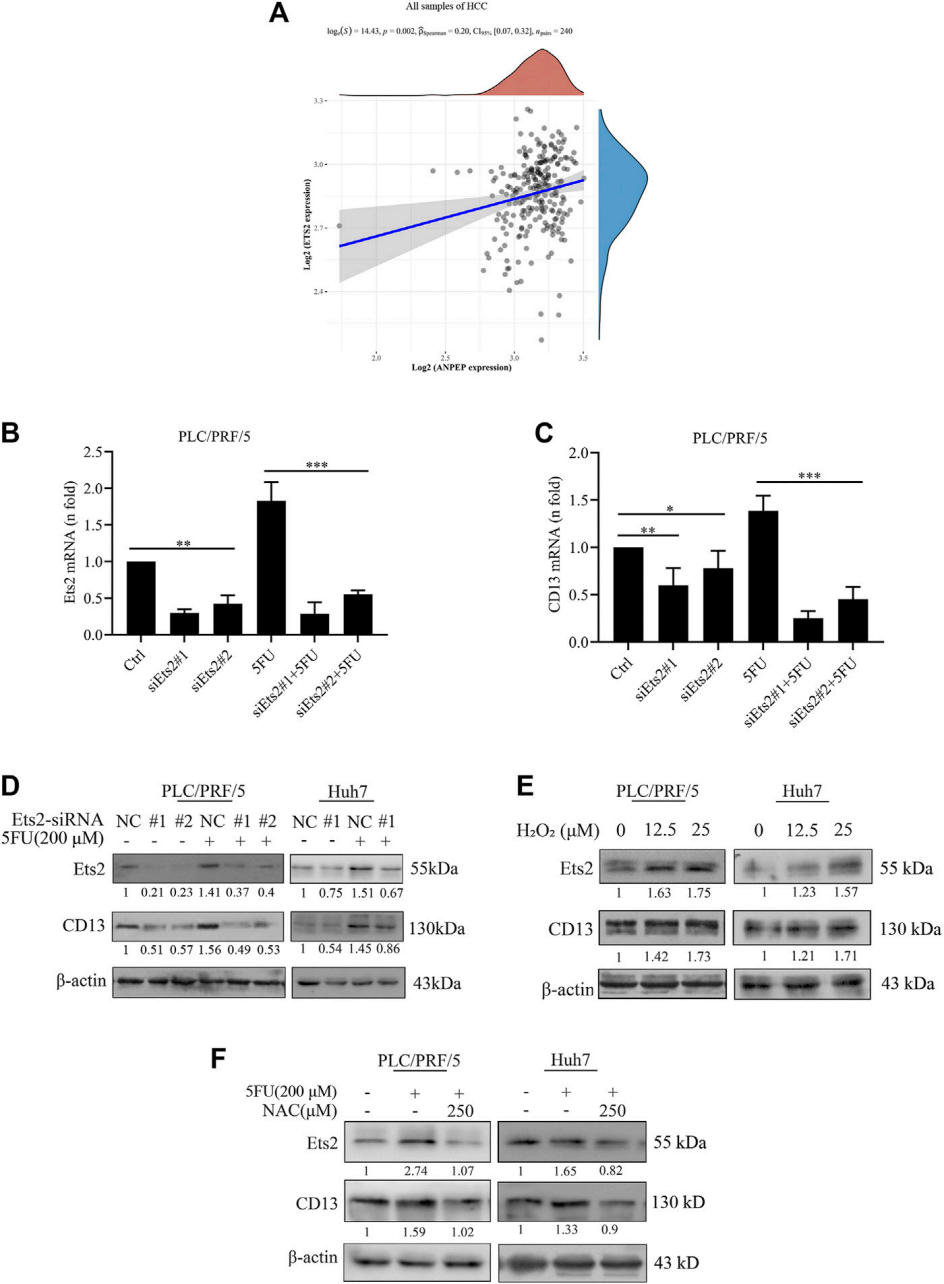
ROS upregulates CD13 expression through activating transcriptional factor Ets2. **(A)** The correlation between the expression of CD13 and Ets2 in liver cancer samples (data from TCGA). ANPEP indicates CD13. **(B,C)** qRT-PCR analysis on PLC/PRF/5 cells transfected with Ets2-siRNA and treated with 5FU(200 μM for 24 h). Data are presented as mean ± SEM (*n* = 3). *p ≤ 0.05, ***p ≤ 0.001. **(D,E)** Immunoblotting on Ets2 and CD13 protein expression PLC/PRF/5 and Huh7 cells. **(D)** Cells transfected with Ets2-siRNA and treated with 5FU for 48 h **(E)** cells treated with H_2_O_2_ (12.5, 25 μM) for 48 h. **(F)** Cells treated with combination of 5FU and NAC. A representative immunoblot from three independent experiments giving similar results is shown and *β*-actin was used as a protein loading control.

Currently we know that chemotherapeutic drug promoted CD13 expression by up-regulating ROS generation, but the key protein of ROS mediated CD13 expression is still not clear. It has been suggested that CD13/APN transcription is induced by RAS/MAPK-mediated phosphorylation of Ets-2 in activated endothelial cells (Petrovic et al., 2003). In order to test whether Ets2 plays a role in the upregulation of CD13 expression by ROS in HCC, we firstly collected hepatocellular carcinoma data sets from the Cancer Genome Atlas (TCGA) data and analyzed the correlation between CD13 and Ets-2 gene expression. The results showed that CD13 expression was positively correlated with Ets-2 (*p* = 0.002, Figure 2A). Then, two Ets2-siRNA sequences were designed and synthesized. The silencing efficacy of Ets2-siRNA in PLC/PRF/5 and Huh7 cells were determined by qPCR and immunoblot. The results showed that Ets2-siRNA#1 has a stronger gene silencing efficacy than Ets2-siRNA#2. CD13 expression was downregulated after Ets2 knocking down (Figures 2B–D), indicating that Ets2 is an upstream signaling molecule of CD13. Next, we examined whether H_2_O_2_ affect the Ets2 and CD13 expression in PLC/PRF/5 and Huh7 cells. The results showed that Ets2 and CD13 expression were significantly increased by H_2_O_2_ treatment (Figure 2E). In addition, we determined the effect of 5FU and NAC combination on the expression of Ets2 and CD13 by immunoblot. The data indicated that the level of Ets2 and CD13 were upregulated after a single 5FU treatment. Meanwhile, the expression of Ets2 and CD13 was downregulated after combination treatment of 5FU and NAC compared to 5FU single (Figure 2F). These results suggested that chemotherapeutic drug activates Ets2 expression through upregulate of ROS and ultimately promotes the expression of CD13.

### CD13 Overexpression Upregulates Reactive Oxygen Species Scavenging Genes Transcription

**FIGURE 3:**
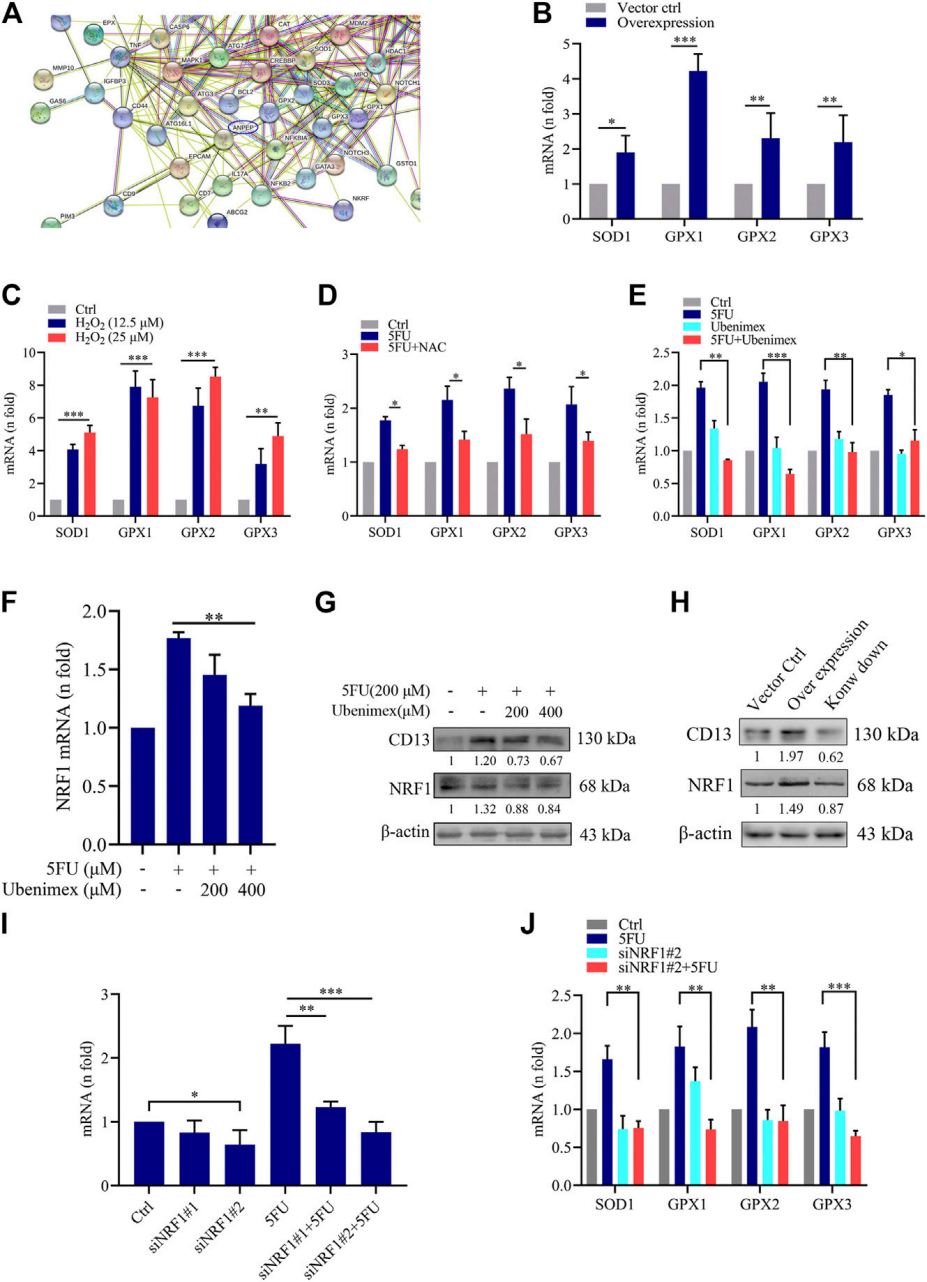
CD13 Overexpression promotes ROS scavenging genes transcription. **(A)** RNA transcriptome chip analysis on the mRNA levels of vector control and CD13 overexpression PLC/PRF/5 cells. ANPEP indicates CD13. **(B)** The mRNA levels of SOD1, GPX1, GPX2 and GPX3 analyzed in vector control and PLC/PRF/5/CD13 cells by qRT-RCR. **(C–F)** qRT-PCR on mRNA levels of ROS scavenging genes in PLC/PRF/5 cells **(C)** treated with H_2_O_2_ (12.5, 25 μM) for 24 h **(D,E)** treated with 5FU (200 μM) combined with NAC (500 μM) or ubenimex (200 μM) **(F)** treated with combination of 5FU and ubenimex. Data are presented as mean ± SEM (*n* = 3). *p ≤ 0.05, ***p ≤ 0.001. **(G)** CD13 and NRF1 expression was determined by using Immunoblot. A representative immunoblot from three independent experiments giving similar results is shown and *β*-actin was used as a protein loading control. **(H)** PLC/PRF/5 cells were infected with lentivirus. After puromycin screening, PLC/PRF/5 cells exhibiting stable CD13 overexpression or knockdown were obtained. Immunoblot was performed to detect CD13 and NRF1 expression. A representative immunoblot from three independent experiments giving similar results is shown and *β*-actin was used as a protein loading control. **(I,J)** qRT-PCR on mRNA expression of NRF1 and ROS scavenging genes in PLC/PRF/5 cells transfected with NRF1-siRNA and treated with 200 μM 5FU for 24 h. Data are presented as mean ± SEM (*n* = 3). *p ≤ 0.05, ***p ≤ 0.001.

To further explore the mechanism of ROS-induced CD13 expression in liver cancer cells, we performed RNA transcriptome gene chip analysis using empty vector control and CD13 overexpression liver cancer PLC/PRF/5 cells. The results showed that there were 1226 genes up-regulated and 764 genes down-regulated with statistically significant difference (Supplementary Figures S1A-C). The relationship between CD13/APN and differential genes were predicted using chip data, it showed that the ROS scavenging genes SOD1, GPX1, GPX2 and GPX3 were closely related to CD13 (Figure 3A). Therefore, we further confirmed the mRNA level of SOD1, GPX1, GPX2 and GPX3 in PLC/PRF/5/CD13 cells using qRT-PCR. The data suggested that SOD1, GPX1, GPX2 and GPX3 were significantly up-regulated in PLC/PRF/5/CD13 cells compared with the vector control cells (Figure 3B). Then, we treated PLC/PRF/5 cells with H_2_O_2_ or 5FU to detect the mRNA level of SOD1, GPX1, GPX2, and GPX3. They were all up-regulated as expected. Meanwhile, the antioxidant NAC and CD13 inhibitor ubenimex blocked the up-regulation of ROS scavenging gene transcription (Figures 3C–E).

Nuclear respiratory factors 1 and 2 (NRF1 and NRF2) is an important transcription factor that regulates the cellular oxidative stress responses and is also the central regulator in maintaining the intracellular redox homeostasis. They can through regulate the expression of a series of antioxidant proteins, keep the cells in a stable state and maintain the redox dynamic equilibrim (Radhakrishnan et al., 2010; Wan et al., 2012; Zhang and Xiang, 2016). In some studies have detected the expression levels of NRF2 in PLC/PRF/5 cells, so we chose to further study NRF1. Firstly, to confirm the relationship between CD13 induced ROS scavenging gene transcription and NRF1, we treated PLC/PRF/5 cells with combination treatment of 5FU and ubenimex. Then the mRNA and protein levels of CD13 and NRF1 were detected by qRT-PCR and immunoblotting. The results showed that the mRNA and protein levels of NRF1 were downregulated after CD13 suppression compared with single 5FU group (Figures 3F,G). In addition, we detected NRF1 expression level when CD13 knockdown and overexpression. As expected, the immunoblotting results showed that NRF1 expression upregulated in PLC/PRF/5/CD13 cells compared with Ctrl cells, and CD13 knockdown downregulated NRF1 expression (Figure 3H), which suggests that NRF1 was located downstream of CD13.

To verify whether NRF1 was related to CD13 induced ROS scavenging gene transcription, two NRF1-siRNA sequences were designed and synthesized, and the silencing efficacy in PLC/PRF/5 cells was detected by qRT-PCR. The results showed that the silence efficacy of siNRF1#2 is more potent (Figure 3I). Then, NRF1 silenced PLC/PRF/5 cells were treated with 5FU and the mRNA levels of SOD1, GPX1, GPX2 and GPX3 were detected by qRT-PCR. The results showed that mNRA levels of ROS scavenging gene were downregulated after NRF1 silencing. 5FU could upregulate the ROS scavenging gene mRNA levels. However, 5FU could not upregulate the ROS scavenging gene mRNA levels again after knocking down NRF1 transcription (Figure 3J). All the above results together indicated that CD13 activates NRF1 to promote the transcription of ROS scavenging gene.

### CD13 Inhibitor Ubenimex Enhances Cytotoxicity of Different Chemotherapy Drugs

**FIGURE 4:**
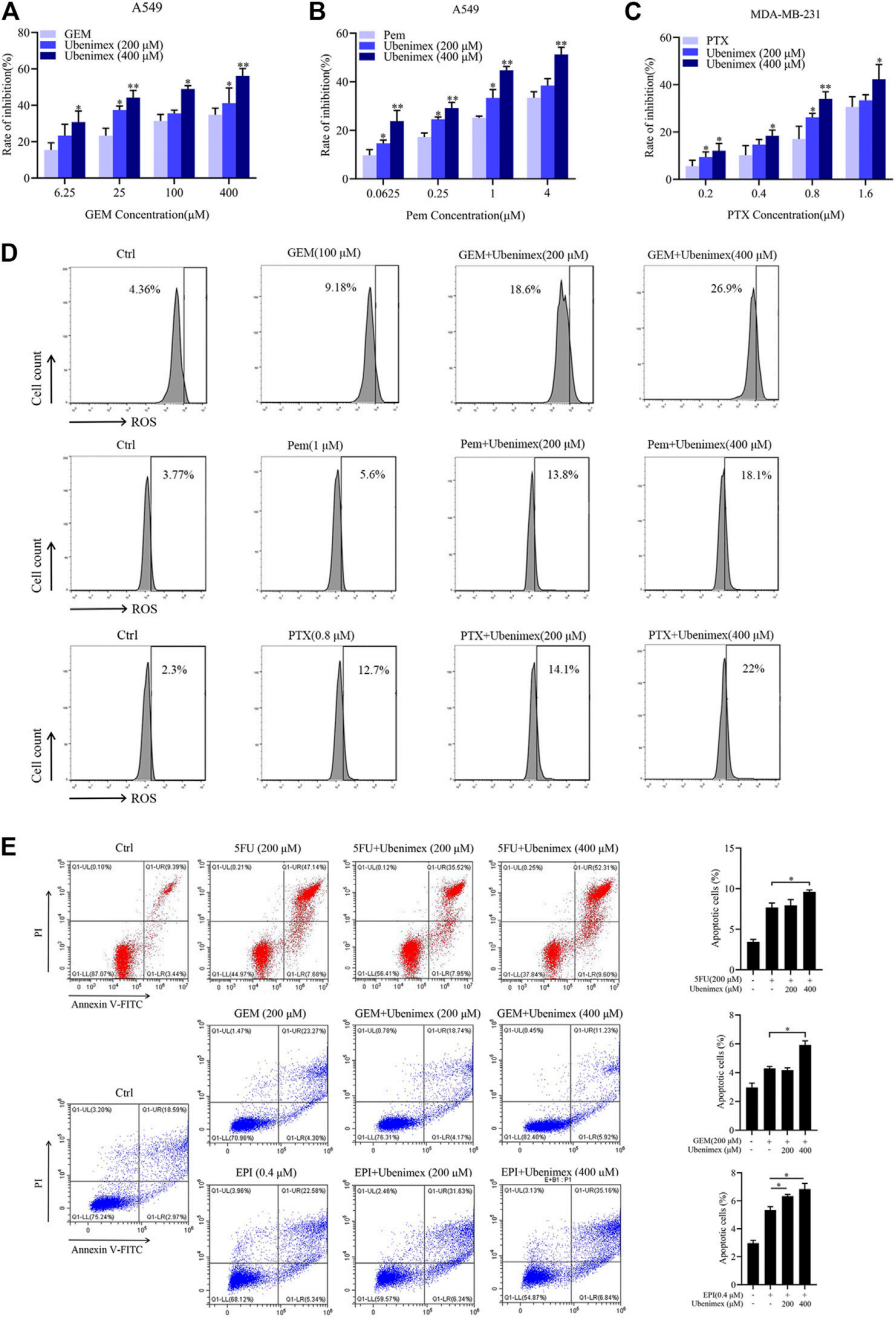
CD13 inhibitor ubenimex enhances cytotoxicity of different chemotherapeutic drugs. **(A–C)** EdU assay on cell proliferation of A549 and MDA-MB-231 cells treated with GEM, Pem and PTX or combined with ubenimex for 48 h. **(D)** A549 and MDA-MB-231 cells were treated with GEM, Pem, and PTX or combined with ubenimex for 48 h, the levels of ROS detected by flow cytometry. A representative ROS level from three independent experiments giving similar results is shown. **(E)** Flow cytometric analysis of cell apoptosis induced by a chemotherapeutic drug and ubenimex combination in PLC/PRF/5 cells using annexinV-FITC/PI. Data are presented as mean ± SEM (*n* = 3), *p ≤ 0.05, **p ≤ 0.01.

From the above data, chemotherapeutic drug could induce ROS generation then promote the expression of CD13. CD13 overexpression could induce reactive oxygen scavenging gene transcription. Therefore, when intracellular ROS rised to a certain levels, CD13 would be upregulated in feedback to promote the transcription of ROS scavenging genes leading to down-regulation of ROS levels. Our previous studies have shown that chemotherapeutic durg combined with CD13 inhibitor can eliminate liver cancer stem cells by increasing intracellular ROS and DNA damage induced by ROS (Dou et al., 2017). Can CD13 inhibitor enhance cytotoxicity of different chemotherapy drugs which usually raise ROS levels. CD13 inhibitor ubenimex was employed to treat human lung adenocarcinoma cell line A549 and human breast cancer cell line MDA-MB-231 cells combined with GEM, pemetrexed (Pem) and paclitaxel (PTX). The cell proliferation ability was tested by EdU assay. The results showed that the cell proliferation ability decreased after the combination treatment of ubinimex and chemotherapeutic durg, compared with single chemotherapy drug group (Figures 4A–C). Meanwhile, the ROS level was detected in two cancer cells by treated with single chemotherapeutic drug and combination therapy, the results showed that the level of ROS was upregulated after combination therapy, compared with single chemotherapeutic drug treatment (Figure 4D). Finally, apoptosis of PLC/PRF/5 cells treated by 5FU, GEM and EPI in combination with ubenimex was detected by flow cytometry. The data showed that apoptosis rate increased significantly after combination treatment of ubenimex and chemotherapeutic drug (Figure 4E). The above results indicated that ubenimex could enhance cytotoxicity of different chemotherapy drugs.

### Reactive Oxygen Species Promote CD13-Dependent Autophagy

**FIGURE 5:**
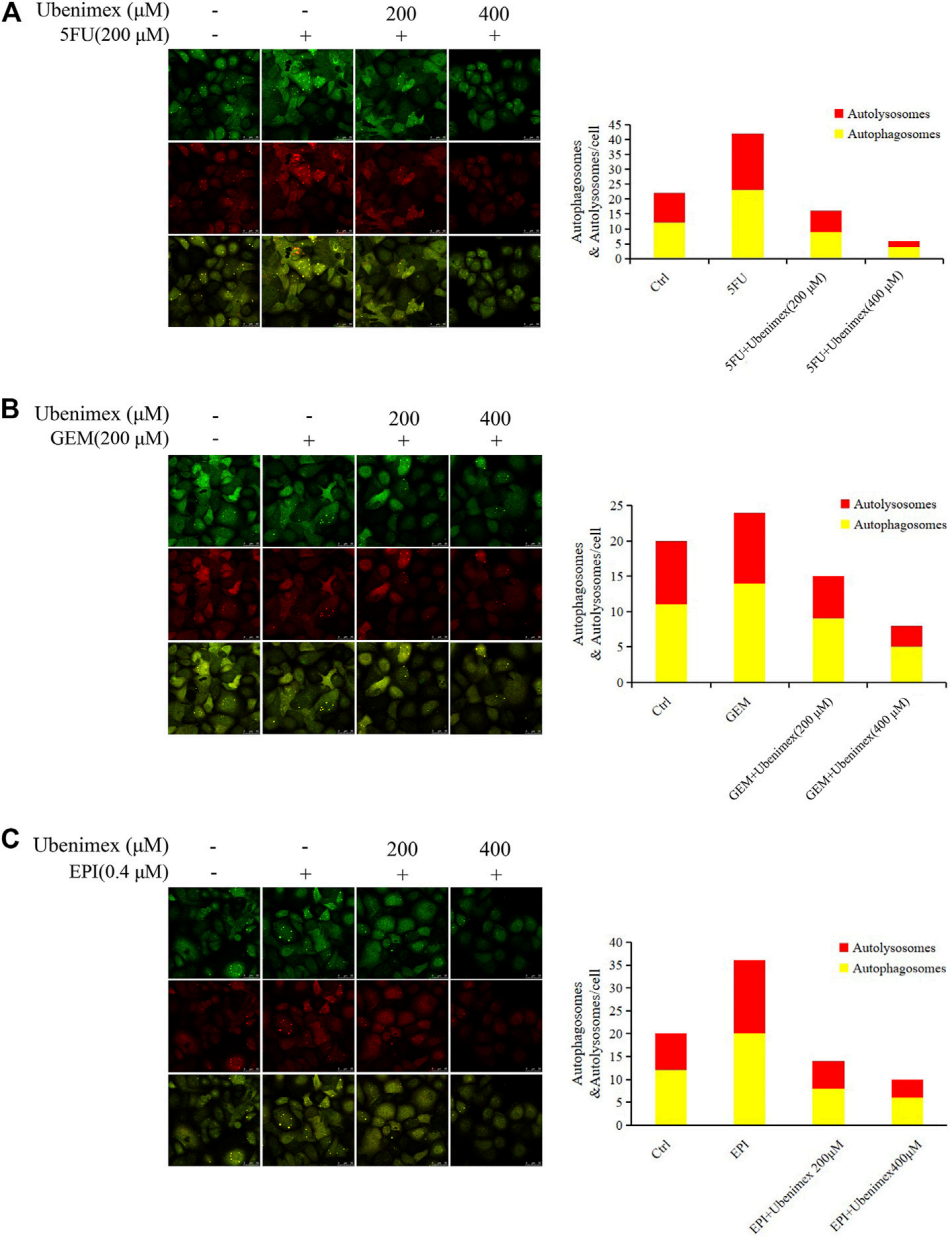
ROS upregulates CD13 expression and induces cell autophagy. **(A–C)** PLC/PRF/5 cells transfected with StubRFP-sensGFP-LC3B lentivirus and treated with 5FU, GEM or EPI alone or in combination with ubenimex. Quantification of autophagosome and autolysosome formation (puncta staining sites) per cell. *p ≤ 0.05, **p ≤ 0.01.

We have previously reported that CD13 induces autophagy through the P38/Hsp27/CREB/ATG7 pathway to promote chemoresistance in hepatocellular carcinoma cells (Zhao et al., 2020). Therefore, we speculated that ROS-mediated CD13 expression could also induce autophagy. Therefore, we treated PLC/PRF/5 cells transfected with StubRFP-sensGFP-LC3B lentivirus by 5FU, GEM and EPI alone or in combination with ubenimex. Then we observed the changes in autophagy through laser confocal microscope. The results showed that all three chemotherapeutic drugs could induce the autophagy process, which could be suppressed by ubenimex (Figures 5A–C). The above results indicated that ROS promotes CD13-dependent autophagy.

### Combination of Ubenimex and Chemotherapeutic Drug Inhibit Mice Tumor Growth *In Vivo*

**FIGURE 6:**
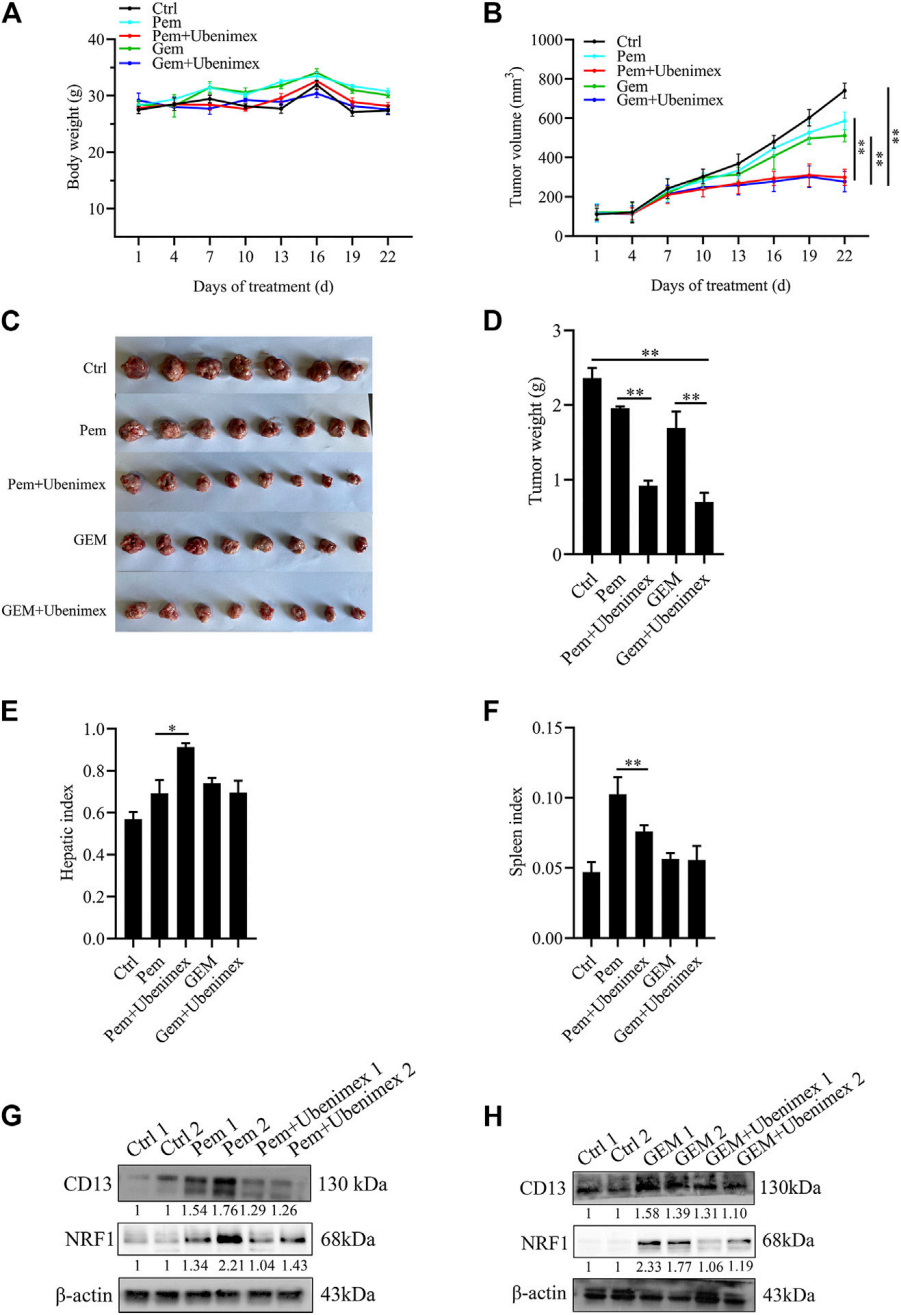
Combination of ubenimex and 5FU suppresses tumor growth *in vivo*. **(A,B)** Body weight and tumor volumes of mice with different drug treatment. **(C–F)** Tumor tissue, liver and spleen tissue of mice were weighed. Data are presented as mean ± SEM. *p ≤ 0.05, **p ≤ 0.01. **(G,H)** Immunoblotting on protein expression in tumors treated with Pem, or GEM, ubenimex + Pem, and ubenimex + GEM. *β*-actin was the protein loading control.

In this study, Kunming mice bearing tumor cells were utilized to confirm whether CD13 suppression enhances the anti-tumor effect of GEM and Pem *in vivo*. Approximately, 1 × 10^^7^ H22 cells were injected into the right armpit of Kunming mice subcutaneously. The results showed that ubenimex combined with GEM or Pem could significantly inhibit the growth of tumor tissue, compared with GEM or Pem alone (Figures 6A–F). We then detected protein expression in the tumor tissue. Immunoblotting results indicated that GEM and Pem could induce the expression of CD13 and NRF1, while ubenimex could suppress the protein expression (Figures 6G,H). In summary, ubenimex could enhance the anti-tumor effect of chemotherapeutic drug and inhibit the growth of tumor cells *in vivo*.

## Dicussion

Hepatocellular carcinoma (HCC) is the most common primary liver cancer, the sixth most common cancer in the world, and the second leading cause of cancer-related deaths (Ferlay et al., 2015). Global morbidity and mortality of HCC is still increasing in the most of countries (Jemal et al., 2011). In China, HCC is ranked as the fourth most common tumor, accounting for more than 90% of all primary liver malignancies (Chen et al., 2016). The prognosis of HCC was dismal with a 3-years survival rate of 12.7% and a median survival of 9 months (Giannini et al., 2015). Although, many treatment options are available for patients with HCC, including surgical resection, local ablation therapy, chemoembolization, liver transplantation and molecular target therapy. Nevertheless, the prognosis of HCC remains poor, due to intrahepatic spread, postsurgical recurrence and chemotherapy resistance (Forner et al., 2012). In the last decade, improved drug therapy drugs have significantly prolonged the survival time of HCC patients with advanced diseases. The commonly used therapeutic regimens include sorafenib, adriamycin (ADM), 5-Fluorouracil (5FU), platinum-containing anti-cancer drugs, camptothecin and Gemcitabine (Kalyan et al., 2015). However, the acquisition of multi-drug resistance (MDR) to these drugs is the Achilles’ heel in clinical oncology, and may result in a poor prognosis. Intrinsic or acquired drug resistance is defined as the resistance of malignant cells to different structurally and functionally unrelated anticancer drugs. The mechanisms of HCC drug resistance are complex and including the increased expression of drug efflux transporters that recognize and pump out anticancer drugs out of tumor cells, redistribution of intracellular drug accumulation of drugs, inactivation of apoptotic signaling pathways, enhanced DNA damage repair ability, accelerated drug metabolism and activation of cancer stem cells (CSCs) (Llovet et al., 2015). However, up to now, the exact mechanisms of drug resistance in hepatocellular carcinoma remain to be investigated.

CD13, a biomarker of human liver CSCs, which is associated with the self-renewal, differentiation potential, signal transduction, drug resistance, recurrence and prognosis of liver cancer stem cells (LCSCs) (Christ et al., 2011; Kim et al., 2012). CSCs possess stem cell-like properties, including the ability for self-renewal and CSCs can start carcinogenesis and are responsible for tumor recurrence after treatment (Shenouda et al., 2020). Our previous studies have confirmed that inhibition of CD13 could increase the sensitivity of HCC cell to 5FU and reverse their drug resistance, thereby indicating that CD13 is potential therapeutic target. In addition, we have also reported that CD13 inhibitor BC-02 impaired the properties of liver CSCs by targeting CD13 and up-regulating the intracellular ROS and ROS-induced DNA damage (Dou et al., 2017). In this study, we further explored the molecular mechanism of chemotherapeutic drugs up-regulating ROS-induced CD13 expression. Our research showed that chemotherapeutic drugs 5FU, GEM and EPI could upregulate intracellular ROS and activate Ets2 to induce CD13 expression. When intracellular ROS raised to a certain level, CD13 is up-regulated in feedback to promote the expression of ROS elimination gene, thus down-regulating the production of ROS.

Autophagy is a multi-step catabolic process that involving initiation (that is phagophore formation), autophagosome biogenesis, lysosome biogenesis, autophagosome–lysosome fusion and lysosomal degradation (Murrow and Debnath, 2013; Shen and Mizushima, 2014). ROS are known to induce autophagy, but the mechanisms underlying this induction are poorly understood (Scherz-Shouval and Elazar, 2011; Li et al., 2015). Therefore, stubRFP-sensGFP-LC3B lentivirus was employd to transfect PLC/PRF/5 cells to labeled LC3-Ⅱ and observed whether inhibition of CD13 could down-regulate autophagy caused by ROS. Our results showed that ubenimex could prevent chemotherapy drugs from up-regulating ROS induced autophagy.

In present study, we confirmed that chemotherapeutic drugs induce CD13 expression by up-regulating ROS levels. CD13 suppression could enhance the cytotoxicity of chemotherapeutic drugs in cells. However, the autophagy process induced by ROS and regulatory mechanisms of signaling pathways, transcriptional factors, and gene expression involved in CSCs are still poorly defined, and further research is required. Understanding these mechanisms may offer advance therapeutic approaches to eradicate, detect, and even prevent cancer.

## Data Availability

The original contributions presented in the study are included in the article/Supplementary Material, further inquiries can be directed to the corresponding authors.
