# Supplementary material for: Suppression of CD13 Enhances the Cytotoxic Effect of Chemotherapeutic Drugs in Hepatocellular Carcinoma Cells
Source: Front Pharmacol. 2021 May 11;12:660377. doi: 10.3389/fphar.2021.660377 (PMC8144446; doi:10.3389/fphar.2021.660377)
Supplement: Supplementary file 1 [file Image1.pdf]

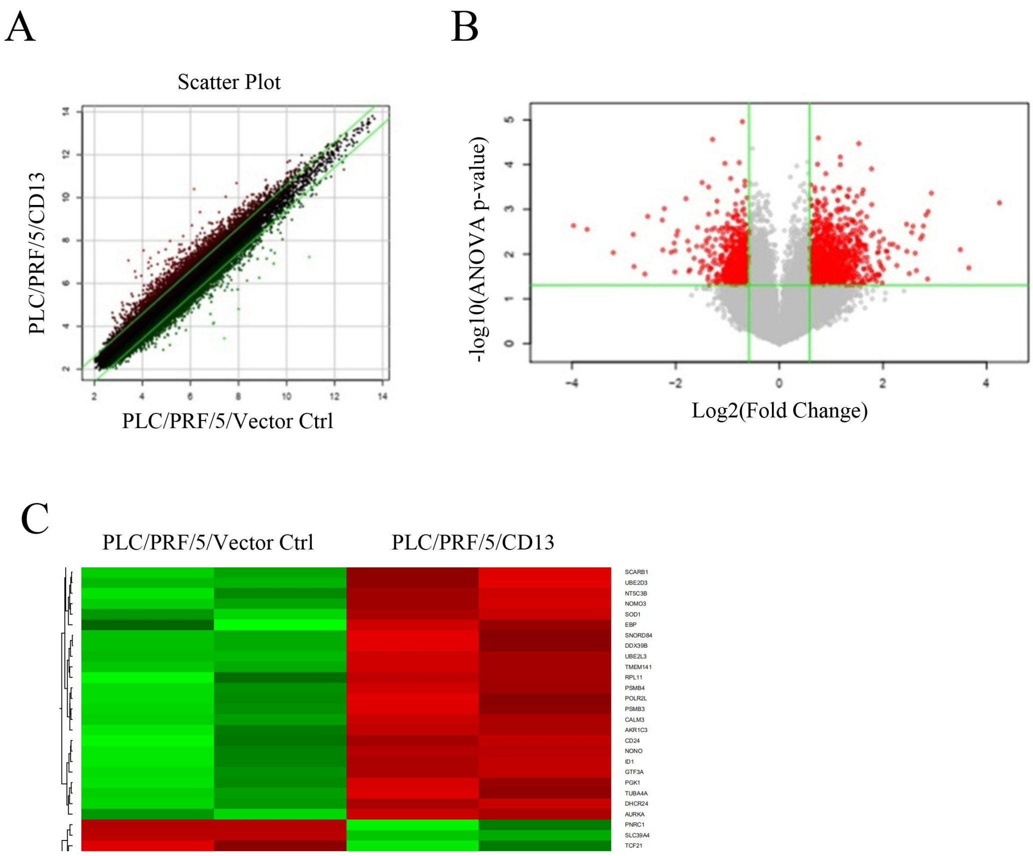

Supplementary Fig 1

Significance statement: Our study demonstrates that chemotherapy agents up-regulate ROS-induced CD13 expression and promotes HCC cell chemoresistance and CD13 inhibitor ubenimex could inhibit this effect and partially revert chemotherapy resistance of HCC. Our results collectively suggest that CD13 may serve as a potential target for overcoming HCC resistance.
